# Anti‐EGFR scFv tetramer (tetrabody) with a stable monodisperse structure, strong anticancer effect, and a long *in vivo* half‐life

**DOI:** 10.1002/2211-5463.12073

**Published:** 2016-05-16

**Authors:** Ryutaro Asano, Noriaki Koyama, Yasuyo Hagiwara, Yosuke Masakari, Ryota Orimo, Kyoko Arai, Hiromi Ogata, Shozo Furumoto, Mitsuo Umetsu, Izumi Kumagai

**Affiliations:** ^1^ Department of Biomolecular EngineeringGraduate School of EngineeringTohoku UniversitySendaiJapan; ^2^ Department of Radiopharmaceutical ChemistryGraduate School of Pharmaceutical SciencesTohoku UniversitySendaiJapan; ^3^ Present address: Department of Biotechnology and Life ScienceGraduate School of EngineeringTokyo University of Agriculture and TechnologyTokyo184‐8588Japan

**Keywords:** anticancer effect, EGFR, ScFv, ScFv multimer, tetrabody

## Abstract

The development of single‐chain variable fragments (scFvs) as therapeutic agents has the potential to reduce the high cost of antibody production, but the development process often impairs scFv functions such as binding affinity and pharmacokinetics. Multimerization is one strategy for recovering or enhancing these lost functions. Previously, we constructed several antiepidermal growth factor receptor (EGFR) scFv multimers by modifying linker length and domain order. Antitumor effects comparable with those of the currently approved anti‐EGFR therapeutic antibodies were observed for scFv trimers. In the present study, we fractionated an anti‐EGFR scFv tetramer from the intracellular soluble fraction of an *Escherichia coli* transformant. Compared with the trimer, the tetramer showed higher affinity, greater cancer cell growth inhibition, and prolonged blood retention time. Furthermore, the tetramer did not dissociate into the trimer or other smaller species during long‐term storage (up to 33 weeks). Thus, our developed scFv tetramer is an attractive candidate next‐generation anti‐EGFR therapeutic antibody that can be produced via a low‐cost bacterial expression system.

**Abbreviations**
BS: bacterial supernatant
EGFR: epidermal growth factor receptor
Fab: fragment antigen‐binding
Fc: fragment crystallizable
h528: humanized anti‐EGFR antibody 528
ICS: intracellular soluble
MTS: 3‐(4,5‐dimethylthiazole‐2‐yl)‐5‐(3‐carboxymethoxyphenyl)‐2‐(4‐sulfophenyl)‐2H‐tetrazolium inner salt
PBS: phosphate‐buffered saline
PP: periplasmic
RU: resonance unit
scFv: single‐chain variable fragment
sEGFR: soluble EGFR
SUV: standardized uptake value
*V*_H_: variable heavy domain
*V*_L_: variable light domain

Although conventional monoclonal antibodies are now used to treat a variety of difficult to cure diseases, such as cancers, their use is limited by their high production costs due to the requirement of a mammalian expression system and their poor penetration into tumor tissue 1, 2. Advances in recombinant technology now make it possible to produce antibody fragments, such as variable fragments and single‐chain variable fragments (scFvs) 3, which, in addition to having high tumor tissue penetration, can be produced via bacterial expression systems, and are therefore ideally suited for large‐scale preparation; however, they are rapidly cleared from the blood, their valency is decreased compared with the parental IgG, and they do not possess a fragment crystallizable (Fc) region. Consequently, variable fragments and scFvs have low target affinity and fail to induce secondary immune functions such as antibody‐dependent cellular cytotoxicity and complement‐dependent cytotoxicity.

Multimerization is one strategy for improving the pharmacokinetics and binding affinity of antibody fragments. In scFvs, the length and composition of the polypeptide linker between the variable heavy (*V*_H_) and light (*V*_L_) domains strongly influences which multimeric structure is formed. For example, a linker comprising 15 amino acid residues leads only to the formation of an scFv monomer, whereas a linker comprising 8–12 residues leads to the formation of scFv dimers known as diabodies. A linker comprising less than five residues, or no linker, leads to the formation of higher order multimers such as trimers (triabodies) and tetramers (tetrabodies) 4, 5, 6, 7, 8. The larger molecular size of the multimers results in a longer serum half‐live and more rapid tumor uptake and blood clearance, which produces better tumor‐to‐blood ratios than those obtained with the parental IgG or the scFv monomer 9. Furthermore, multimerization generally results in multivalency against the target antigen, which not only produces a greater binding affinity due to an avidity effect but also improves function, which may compensate for the lack of induction of secondary immune functions. Even though the binding valencies of the parental IgG and scFv diabody are identical, there are reports that conversion into the diabody can confer a strong agonist activity in the diabody even if the parental IgG has little to no agonist activity, which may be attributable to changes in accessibility to, or the achievable proximity of, target antigens 10, 11.

Epidermal growth factor receptor (EGFR) is an important target for cancer therapeutic agents because it is expressed in many types of solid tumor and its expression level is correlated with malignancy, metastatic phenotype, and poor prognosis 12, 13, 14. Two anti‐EGFR therapeutic antibodies, cetuximab and panitumumab, are currently approved by the US Food and Drug Administration, and other anti‐EGFR therapeutic antibodies are undergoing clinical trials 15. Panitumumab is characterized by its high affinity to EGFR and is thought to function primarily through the blocking of ligand–receptor interactions. It also has a decreased likelihood of damaging normal EGFR‐positive cells because it belongs to the IgG2 subclass and therefore has reduced effector functions compared with cetuximab, which belongs to the IgG1 subclass 15, 16.

Previously, we constructed scFv multimers using humanized anti‐EGFR IgG 528 17. These h528 scFv multimers were produced via a bacterial expression system, and after fractionation they inhibited tumor cell growth by blocking the EGFR phosphorylation in a multimerization‐dependent manner. Among the multimers examined, h528 scFv trimer with the V_H_–V_L_ domain order and no linkers (HLG0 trimer) showed the highest *in vitro* and *in vivo* anticancer effects, which were comparable with those of cetuximab and panitumumab.

In this study, we successfully fractionated h528 scFv HLG0 tetramer and improved the yield obtained with our bacterial expression system. Compared with h528 scFv HLG0 trimer, h528 scFv HLG0 tetramer showed higher affinity, greater tumor cell growth inhibition, and prolonged blood retention time. Furthermore, the tetramer did not dissociate into the trimer or other smaller species during long‐term storage. To our knowledge, this is the first report of the production of a highly stable scFv tetramer that inhibits tumor cell growth; therefore, this tetramer is an attractive candidate next‐generation anti‐EGFR therapeutic antibody that can be produced via a low‐cost bacterial expression system.

## Results

### Preparation of h528 scFv HLG1 dimer from intracellular soluble fraction of an *Escherichia coli* transformant

**Figure 1:**
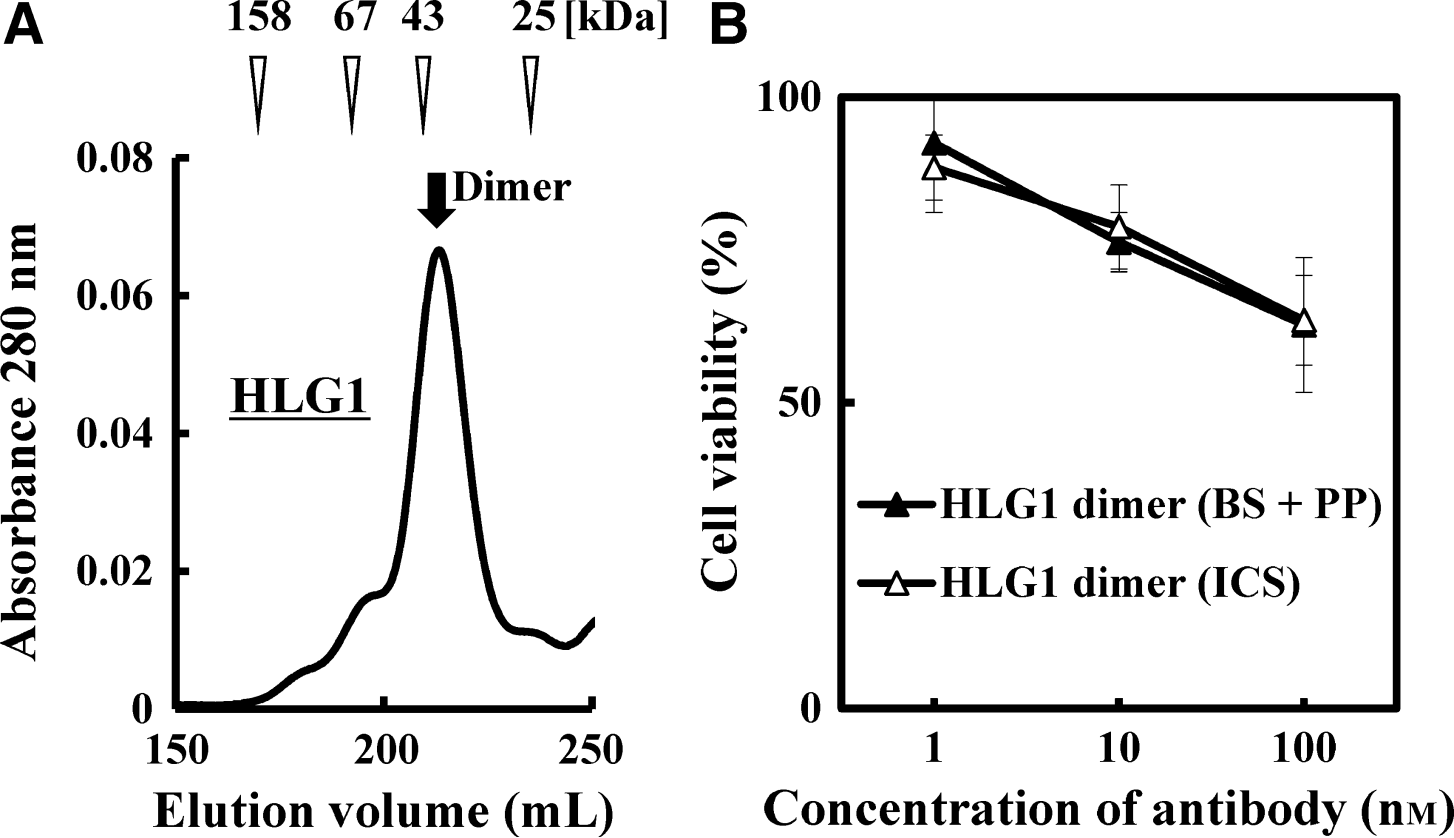
(A) h528 scFv in the *V*_H_–*V*_L_ order with a six‐amino acid linker (HLG1) was expressed in an *Escherichia coli* transformant and purified from intracellular soluble (ICS) fraction using immobilized metal ion affinity chromatography. Gel‐filtration chromatography with a HiLoad 26/600 Superdex 200 pg column was used for further purification. (B) Comparison of the inhibitory effects of HLG1 dimers prepared from ICS or from bacterial supernatant plus periplasmic fractions (BS + PP) on the viability of A431 human epidermoid carcinoma cells. A431 cells were treated for 96 h with different concentrations of antibodies, and then cell viability was determined using the MTS assay. Data are presented as mean ± 1 SD and are representative of at least two independent experiments.

To examine whether greater yields of anti‐EGFR scFv multimers could be obtained from a bacterial expression system, we prepared h528 scFv in the *V*_H_–*V*_L_ order with a six‐amino acid linker (HLG1) from the intracellular soluble (ICS) fraction of an *Escherichia coli* transformant expressing the scFv. Gel‐filtration chromatography after immobilized metal ion affinity chromatography purification showed that HLG1 prepared from ICS fraction predominantly formed dimers, as did the HLG1 prepared previously from secretion fraction (bacterial supernatant plus periplasmic fraction) (Fig. 1A) 17. The final yield of HLG1 dimer prepared from ICS fraction was 0.70 mg·L^‐1^ culture, which was fourfold of that obtained from secretion fraction.

We next compared the inhibitory effects of the HLG1 dimer prepared from ICS and that prepared from the secretion fraction on the growth of human tumor cells using the 3‐(4,5‐dimethylthiazole‐2‐yl)‐5‐(3‐carboxymethoxyphenyl)‐2‐(4‐sulfophenyl)‐2*H*‐tetrazolium inner salt (MTS) assay. Both types of HLG1 dimers showed very similar inhibitory effects on cancer cell viability (Fig. 1B), indicating that preparation from ICS fraction is an ideal strategy for producing anti‐EGFR h528 scFv multimers at high yields.

### Preparation of h528 scFv HLG0 trimer and tetramer

**Figure 2:**
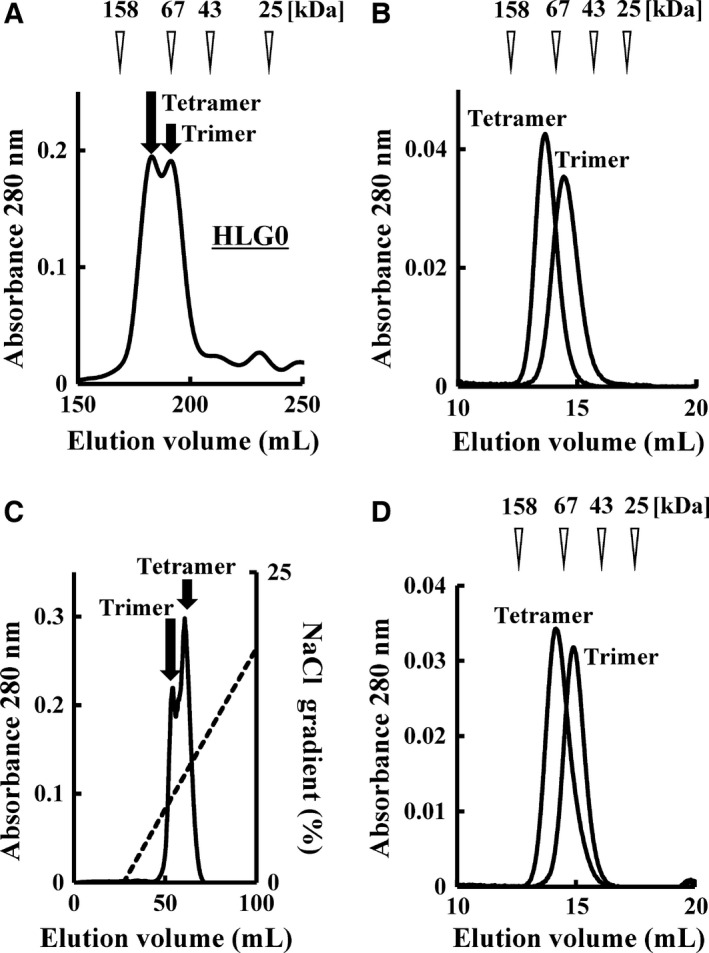
Preparation of h528 scFv HLG0 trimer and tetramer. HLG0 was expressed in an *Escherichia coli* transformant and purified from ICS fraction by means of immobilized metal ion affinity chromatography. Gel‐filtration chromatography with a HiLoad 26/600 Superdex 200 pg column (A) or cation‐exchange chromatography with a Resource S column (C) was used for further purification. The two HLG0 peaks were then fractionated and their monodispersity was confirmed by means of repeated gel‐filtration chromatography after gel‐filtration chromatography (B) or cation‐exchange chromatography (D).

Next, we applied our ICS preparation method to the production of HLG0 trimer and tetramer because we previously confirmed that HLG0 prepared from secretion fraction predominantly formed trimers and that h528 scFv multimers inhibited cancer growth in a multimerization‐dependent manner 17. Two equivalent peaks, which were attributed to the trimer and tetramer species, were observed on the gel‐filtration chromatogram (Fig. 2A). These two peaks were then fractionated and their monodispersity was confirmed by means of repeated gel‐filtration chromatography (Fig. 2B). The final yields of HLG0 trimer and tetramer prepared from ICS fraction were both 0.35 mg·L^‐1^, which was twofold larger than the yield of HLG0 trimer produced from secretion fraction (fourfold if the tetramer is included). The trimer and tetramer were also successfully fractionated by means of cation‐exchange chromatography followed by gel‐filtration chromatography (Fig. 2C,D, respectively). Together, these results showed that HLG0 can form two distinct molecular species (trimer and tetramer), and that preparation from ICS fraction produced a greater amount of tetramers than preparation from secretion fraction.

### Evaluation of binding kinetics by means of surface plasmon resonance spectroscopy

**Figure 3:**
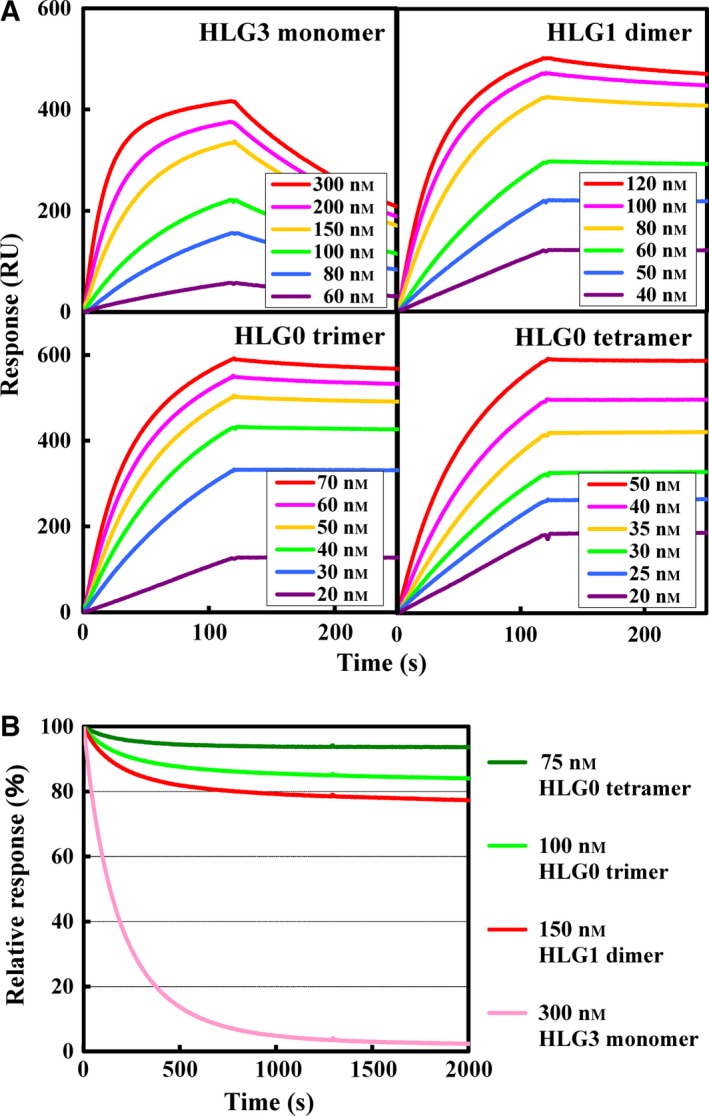
Surface plasmon resonance sensorgrams for h528 scFv species. (A) Soluble epidermal growth factor receptor (sEGFR) was immobilized on a CM5 sensor chip up to 3848 resonance units (RU) and various concentrations of scFv multimers were then allowed to flow over the bound sEGFR at a flow rate of 20 μL·min^‐1^ at 25 °C. The data were referenced by subtracting the response of a blocked blank cell. (B) Comparison of the dissociation behavior of scFv multimers at concentrations equivalent to 300 nm scFv over 2000 s.

To evaluate the apparent affinities of fractionated HLG0 trimer and tetramer, we determined their binding kinetics using immobilized soluble EGFR (sEGFR) and surface plasmon resonance spectroscopy, and then compared them with those of h528 scFv in the *V*_H_–*V*_L_ order with a 16‐amino acid linker (HLG3 monomer), HLG1 dimer, and the approved anti‐EGFR IgG cetuximab (Fig. 3A, Table 1). The resultant sensorgrams showed similar association phases but dissociation phases that were slower with increasing multimerization. The lowest dissociation rate was observed for HLG0 tetramer, and this decrease contributed to a dissociation constant that was lower than that of both HLG0 trimer and cetuximab (Table 1). However, these values were unreliable due to the detection limit of the surface plasmon resonance instrument. Therefore, we next followed the dissociation behavior for 2000 s using the scFv multimers at concentrations equivalent to 300 nm HLG3 monomer (e.g., 75 nm tetramer is equivalent to 75 × 4 = 300 nm monomer). The relative responses at the endpoint showed that higher remained bound to the sensor chip was also multimerization dependent (Fig. 3B, Table 1), indicating that HLG0 tetramer is a distinct molecular species with a different affinity to that of HLG0 trimer.

**Table 1:** Binding and pharmacokinetic parameters of humanized anti‐EGFR antibody 528 scFvs. Kinetic parameters were calculated by means of a global fitting analysis with the assumption of a 1:1 Langmuir binding model

	*k*_on_ (× 10^5^ m ^−1^·s^−1^)	*k*_off_ (× 10^−3^ s^−1^)	*K*_D_ (× 10^−8^ m)	Relative response at 2000 s (%)	Area under the curve (1.5–8 h)
HLG3 monomer	1.4	5.1	3.5	2.4	5.6^a^
HLG1 dimer	2.3	0.28	0.12	77	14.1^a^
HLG0 trimer	3.5	0.14	0.040	84	22.1^a^
HLG0 tetramer	3.0	0.00012	0.000041	94	30.2
Cetuximab	3.0	0.0027	0.00090	n.d.	39.7

EGFR, epidermal growth factor receptor, n.d., not determined, scFv, single‐chain variable fragment.

a Data from our previous report 17.

### Inhibitory effect of h528 scFv HLG0 tetramer on tumor cell growth

**Figure 4:**
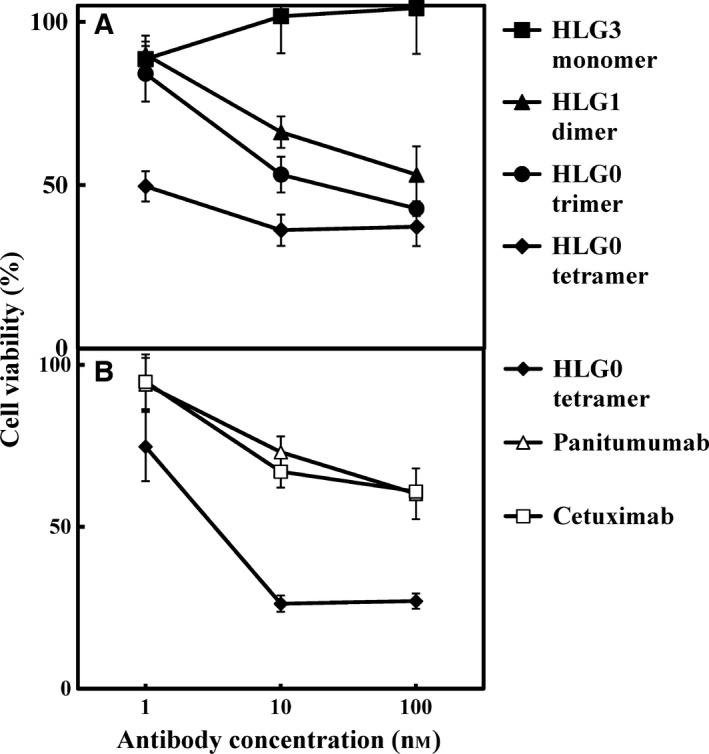
Inhibition of the growth of A431 human epidermoid carcinoma cells by various h528 scFvs. A431 cells were treated for 96 h with different concentrations of antibodies and cell viability was determined by means of the MTS assay. Data are presented as mean ± 1 SD and are representative of at least three independent experiments.

We previously reported that h528 scFvs inhibited the growth of cancer cells in a multimerization‐dependent manner, and that the greatest inhibition was observed for HLG0 trimer 17. Here, we evaluated the inhibitory effect of HLG0 tetramer on the growth of tumor cells, and compared it with those of an h528 scFvs monomer, dimer, and trimer, and also with those of panitumumab and cetuximab using the MTS assay. Although HLG1 dimer and HLG0 trimer both inhibited cancer growth more than the HLG3 monomer, which was consistent with previous results 17, HLG0 tetramer had the greatest overall inhibitory effect (Fig. 4A). Furthermore, the inhibitory effect of HLG0 was also greater than that of panitumumab or cetuximab (Fig. 4B). These results show that the HLG0 tetramer is a novel anticancer antibody that can be prepared by via a low‐cost bacterial expression system.

### Confirmation of the stability of h528 scFv HLGO tetramer and trimer

**Figure 5:**
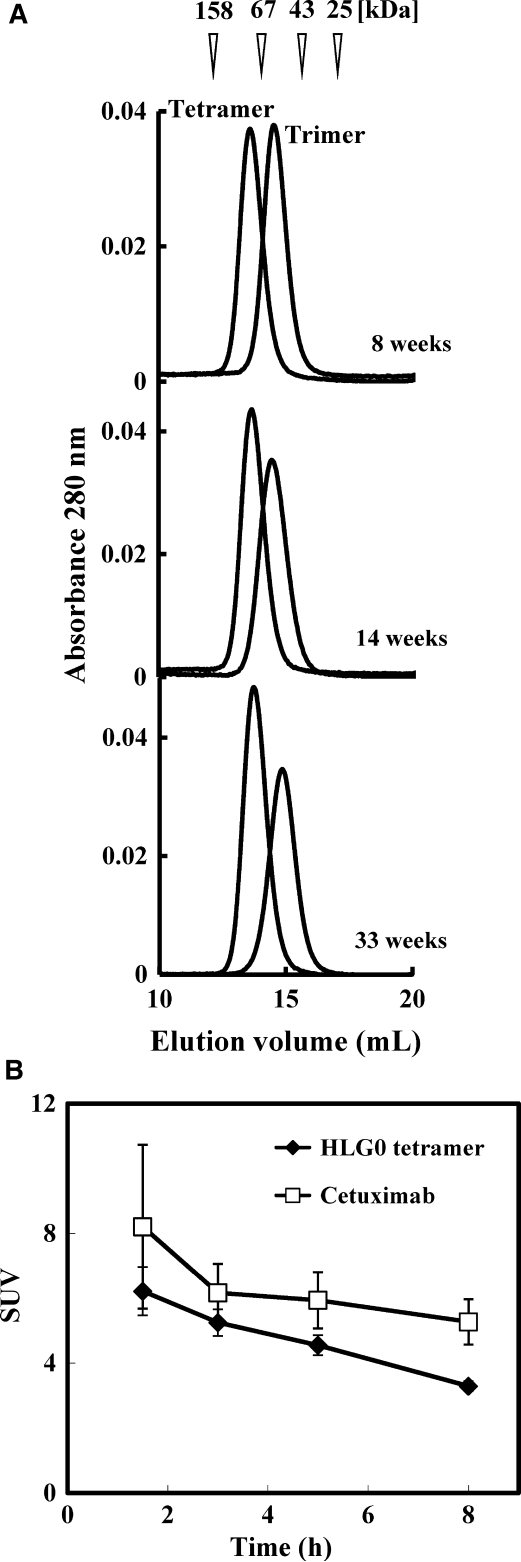
(A) Stability of h528 scFv multimers during long‐term storage as assessed by gel‐filtration chromatography. Fractionated h528 scFvs were applied to a Superdex 200 10/300 GL column after storage for the indicated weeks at 4 °C. (B) Comparison of the clearance from the blood of ^125^I‐labeled HLG0 tetramer and ^125^I‐labeled cetuximab in mice. Imprinting control region mice (*n* = 5) were injected with one of the ^125^I‐labeled antibodies and blood samples were collected from the tail vein at the indicated time points. Standardized uptake value (SUV) = (Blood radioactivity/Blood weight)/(Injected radioactivity/Body weight).

It is crucial that therapeutic recombinant proteins remain structurally stable over time; however, there is a concern that scFv multimers made through noncovalent association dissociate during long‐term storage or when diluted after administration. Therefore, by means of gel‐filtration chromatography, we evaluated the stability of fractionated HLG0 trimer and tetramer that had been stored for 8, 14, or 33 weeks. A single, monodisperse peak at each time point was obtained for both species, indicating that once fractionated, both multimeric structures remained stable during long‐term storage (Fig. 5A).

Next, we compared the *in vivo* stabilities of HLG0 tetramer and cetuximab by measuring the area under the curve by means of radioiodine labeling. Compared with data from a previous report, 17 the area under the curve_(1.5–8 h)_ for HLG0 tetramer was increased 5.4‐fold compared with that for HLG3 monomer, 2.1‐fold compared with that for HLG1 dimer, and 1.4‐fold compared with that for HLG0 trimer; however, it was decreased 1.3‐fold compared with that for cetuximab (Table 1). These values correlated with calculated molecular weight and suggested that the stability of HLG0 tetramer is sufficient for it to be used *in vivo* as a therapeutic agent.

## Discussion

Previously, we developed next‐generation anti‐EGFR × anti‐CD3 bispecific antibodies that included the human Fc region, and studies to assess their clinical usefulness are currently underway 18, 19, 20. However, the recruitment of T cells by bispecific antibodies has prompted concerns about adverse effects on the central nervous system, which have led to the permanent discontinuation of certain investigative studies 21. In addition, although IgG‐like bispecific antibodies are an attractive format, they are difficult to express in bacteria, which leads to high production costs. More importantly, only a few engineered antibodies or antibody fragments had been approved by the US Food and Drug Administration; it therefore remains unknown which recombinant format and strategy is more promising and further development is needed.

Multimerization of antibody fragments is one strategy for improving the drawbacks associated with antibody fragments such as rapid clearance, decreased valence, and lack of an Fc region. There have also been reports of the induction of strong agonist activities by converting to the scFv diabody from the parental IgG 10, 11. Among the two currently approved therapeutic anti‐EGFR antibodies, panitumumab is characterized by its high affinity, and it does not induce natural killer cell‐mediated antibody‐dependent cellular cytotoxicity like cetuximab does 15, 16. We previously constructed anti‐EGFR scFv multimers that inhibited cancer cell growth in a multimerization‐dependent manner 17. Among these multimers, HLG0 trimer showed the highest *in vitro* and *in vivo* antitumor effects, which were comparable with those of cetuximab and panitumumab.

In this study, we increased the yield of scFv multimers obtained by preparing them from ICS fraction of an *E. coli* transformant instead of from secretion fraction, and HLG1 dimers prepared from the two fractions had comparable tumor cell growth inhibitory effects (Fig. 1A,B). When HLG0 was expressed, two equivalent peaks representing the trimer and tetramer were observed on gel‐filtration chromatogram and cation‐exchange chromatogram (Fig. 2A,C), and the monodispersity of these two species was confirmed by means of repeated gel‐filtration chromatography (Fig. 2B,D). Compared with HLG0 trimer, HLG0 tetramer showed higher affinity (Fig. 3, Table 1), greater cancer cell growth inhibition (Fig. 4), and prolonged blood retention time (Table 1). Furthermore, HLG0 tetramer did not dissociate into the trimer or other smaller species during long‐term storage (Fig. 5A). Concentrating each fractionated multimer using an ultrafiltration device also did not affect their multimeric structures, as confirmed by means of repeated gel‐filtration chromatography (data not shown). Together, these results indicate that HLG0 tetramer and trimer have distinct, intrinsically stable, and separable molecular structures.

To date, several alternative binding scaffolds have been developed 22, 23, among which, the designed ankyrin repeat proteins (DARPins) are particularly promising 24. DARPins can be expressed in the cytoplasm of *E. coli*, and recently the inhibitory effects on tumor cell growth of DARPins targeting EGFR were reported together with a detailed inhibitory mechanism 25. In contrast to DARPins, scFv multimers have the advantage that they can be prepared from already existing, well‐characterized scFvs or directly from IgG. The ability of scFv multimers to exhibit useful functions, such as multivalent binding and cross‐linking of adjacent surface receptors, depends on the flexibility of the linker between the variable fragments and the density and orientation of the antigen‐binding sites, as well as the domain order, type of variable fragment, and structure of the target receptor 26. For example, anti‐CD19 scFv trimers are predominantly inactive and show only monovalent binding to the target cell surface 26, 27, anti‐Lewis^y^ scFv trimers rapidly dissociate into inactive monomers until equilibrium is reached 6, and anti‐HER2 scFv tetramers built using self‐associating peptide show no higher avidity than their dimers 28. In contrast, anti‐EGFR scFv multimers, including the tetramers presented in this study, exert their functions in a precise, multimerization‐dependent manner, suggesting that all of the possible binding sites of the anti‐EGFR scFv multimers are active and that EGFR is an ideal target for specific scFv multimer‐based therapeutics. Note, however, that differences in the domain order of anti‐EGFR scFv multimers did affect their function 17, and therefore different results are likely to be observed with other anti‐EGFR antibody clones.

Multimerization of scFvs is influenced not only by structural factors, such as linker length, domain order, and interaction between V_H_ and V_L_, but also by environmental conditions such as ionic strength, pH, and concentration within the bacterial transformant 26, 29. Although it is unknown why a higher yield of tetramer was obtained from ICS fraction compared with secretion fraction, the increased local concentration in the periplasmic fraction or certain components present in BugBuster reagent may affect the transition of the monomer into the trimer or tetramer. However, concentrating the trimer by means of ultrafiltration did not induce association into tetramers, and vice versa. Although the yields of tetramers thus far obtained are insufficient for further application, large‐scale fermentation of scFv multimers has been reported 30. Additionally, significant alterations of the scFv trimer and tetramer equilibrium ratio have been established via *in silico* modeling‐based mutagenesis to remove steric interference 31. We are now working to increase the yield and transition control of h528 scFv tetramer.

In the present work we assume that the scFv tetramers are active against the EGFR pathway on the basis of a previous study which identified trimers as effective among multimers studied 17. Although this and further data for the tetramers such as *in vivo* therapeutic studies and biodistribution are needed in future work, the present study demonstrated that h528 scFv tetramer has a stable structure, high inhibitory effect on tumor cell growth, and long blood retention time, and is therefore an attractive, low‐cost candidate next‐generation anti‐EGFR therapeutic antibody.

## Materials and methods

### Preparation of anti‐EGFR h528 scFv multimers

Bacterial expression vectors for h528 scFv in the *V*_H_–*V*_L_ order with a 16‐amino acid linker (HLG3), with a 6‐amino‐acid linker (HLG1), and without a linker (HLG0) were constructed previously 17. The scFvs were expressed individually in *E. coli* strain BL21(DE3) (Life Technologies, Carlsbad, CA, USA) and purified from bacterial supernatant plus periplasmic fraction using immobilized metal ion affinity chromatography 32. The scFvs were also prepared from ICS fraction using BugBuster reagent (Merck KGaA, Darmstadt, Germany) according to the manufacturer's instructions. Gel‐filtration chromatography with a HiLoad 26/600 Superdex 200 pg column (GE Healthcare Bio‐Science, Piscataway, NJ, USA) was used for further purification. The column was equilibrated with phosphate‐buffered saline (PBS), and then 5 mL of purified scFv was loaded onto the column at a flow rate of 2.0 mL·min^‐1^. Cation‐exchange chromatography with a Resource S column (GE Healthcare Bio‐Science) was also used for further purification. Purified scFv was loaded onto the column in 50 mm NaH_2_PO_4_ (pH 6) and eluted with a 0–1.0 m NaCl gradient in the same buffer. Gel‐filtration chromatography with a Superdex 200 10/300 GL column (GE Healthcare Bio‐Science) was used to confirm the monodispersity of each fractionated HLG0 species, and to evaluate their stability during long‐term storage. For these experiments, the column was equilibrated with PBS, and then 0.25 mL of scFv was loaded onto the column at a flow rate of 0.5 mL·min^‐1^.

### Cell lines

A431 human epidermoid carcinoma cells were obtained from the Cell Resource Center for Biomedical Research, Institute of Development, Aging, and Cancer, Tohoku University (Sendai, Japan) and cultured in RPMI 1640 medium supplemented with 10% fetal bovine serum, 100 U·mL^‐1^ penicillin, and 100 μg·mL^‐1^ streptomycin.

### *In vitro* growth inhibition assay

*In vitro* growth inhibition of cancer cells was assayed with a MTS assay kit (CellTiter 96 AQueous Non‐Radioactive Cell Proliferation Assay; Promega, Madison, WI, USA) as reported 18, 33, 34. In brief, A431 cells (2000 cells in 200 μL of culture medium containing 0.5% fetal bovine serum) were added to a 96‐well plate and incubated overnight to allow the cells to adhere to the well. After the culture medium was removed by aspiration, scFvs or control IgG were added to each well and the cells incubated for a further 96 h at 37 °C. Each well was then washed three times with PBS and detection reagent was added to each well. Cell viability was calculated according to the following equation: percentage cell viability = [(*A*_490_ of experiment − *A*_490_ of background)/(*A*_490_ of control − *A*_490_ of background)] × 100.

### Surface plasmon resonance spectroscopy

The interactions between sEGFR and the scFvs were analyzed by means of surface plasmon resonance spectroscopy (Biacore 2000, GE Healthcare Bio‐Science). The methods for the expression and purification of sEGFR have been described previously^35^. Briefly, sEGFR was immobilized on a CM5 sensor chip up to 3848 resonance units (RU). Various concentrations of scFv multimers in PBS containing 0.005% Tween 20 were then allowed to flow over the bound sEGFR at a flow rate of 20 μL·min^‐1^ at 25 °C. The surface was regenerated between experiments with 10 mm glycine–HCl (pH 2.0) with no loss of activity. The data were referenced by subtracting the response of a blocked blank cell. BIAevaluation software (GE Healthcare Bio‐Science) was used to analyze the data. Kinetic parameters were calculated by means of a global fitting analysis with the assumption of a 1:1 Langmuir binding model.

### Radiolabeling of antibody

An Iodogen tube was prepared by coating a 1.5‐mL microfuge tube with Iodogen (100 μg per tube, Thermo Fisher Scientific, Waltham, MA, USA), which was then used to radiolabel antibodies with [^125^I]NaI (74 MBq 0.1 mL^‐1^; PerkinElmer, Waltham, MA, USA). Antibodies (600–900 μL, 89–108 μg) and [^125^I]NaI (20–25 μL, 26–37 MBq) were placed in the Iodogen tube and incubated for 15 min at room temperature with vortex mixing. ^125^I‐labeled antibody was then separated from unreacted [^125^I]NaI by means of size‐exclusion chromatography with a Bio‐Gel P‐6 Desalting Cartridge (10 mL, Bio‐Rad, Hercules, CA, USA), eluting with PBS–Tween 20 (0.05%) at a flow rate of 1.5 mL·min^‐1^. Radiochemical purities of the isolated antibodies ranged from 94% to 98%.

### Blood clearance study

The Ethics Committee for Experimental Research in Animals of Tohoku University (Sendai, Japan) approved the study protocol. Male imprinting control region mice (6 weeks old, 27–31 g) were injected in the lateral tail vein with ^125^I‐labeled antibody (2 μg, 150–180 kBq) in PBS solution (0.2 mL). Approximately 10 μL of blood was collected from the contralateral tail vein at 1.5, 3, 5, and 8 h post injection (*n* = 5 at each time point). Radioactivity and weight of the blood were measured with a gamma counter (AccuFLEXγ7000; Hitachi Aloka Medical, Tokyo, Japan). Blood radioactivity is expressed as the standardized uptake value (SUV), which was defined as follows: SUV = (Blood radioactivity/Blood weight)/(Injected radioactivity/Body weight).

## Author contributions

RA, MU, IK, and SF planned experiments. NK, YH, YM, RO, KA, HO, and SF performed experiments. RA, MU, IK, and SF analyzed data. RA and SF wrote the paper.

## References

[1] Zhou XK , Qiu J , Wang Z , Huang NY , Li XL , Li Q , Zhang YB , Zhao CJ , Luo C , Zhang NN *et al* (2012) In vitro and in vivo anti‐tumor activities of anti‐EGFR single‐chain variable fragment fused with recombinant gelonin toxin. J Cancer Res Clin Oncol 138, 1081–1090. 22392077 10.1007/s00432-012-1181-7 PMC11824807

[2] Carter PJ (2006) Potent antibody therapeutics by design. Nat Rev Immunol 6, 343–357. 16622479 10.1038/nri1837

[3] Stigbrand T , Ahlstrom KR , Sundstrom B , Makiya R and Stendahl U (1993) Alternative technologies to generate monoclonal antibodies. Acta Oncol 32, 841–844. 8305234 10.3109/02841869309096144

[4] Pei XY , Holliger P , Murzin AG and Williams RL (1997) The 2.0‐A resolution crystal structure of a trimeric antibody fragment with noncognate VH‐VL domain pairs shows a rearrangement of VH CDR3. Proc Natl Acad Sci U S A 94, 9637–9642. 9275175 10.1073/pnas.94.18.9637 PMC23241

[5] Dolezal O , Pearce LA , Lawrence LJ , McCoy AJ , Hudson PJ and Kortt AA (2000) ScFv multimers of the anti‐neuraminidase antibody NC10: shortening of the linker in single‐chain Fv fragment assembled in V(L) to V(H) orientation drives the formation of dimers, trimers, tetramers and higher molecular mass multimers. Protein Eng 13, 565–574. 10964986 10.1093/protein/13.8.565

[6] Power BE , Doughty L , Shapira DR , Burns JE , Bayly AM , Caine JM , Liu Z , Scott AM , Hudson PJ and Kortt AA (2003) Noncovalent scFv multimers of tumor‐targeting anti‐Lewis(y) hu3S193 humanized antibody. Protein Sci 12, 734–747. 12649432 10.1110/ps.0228503 PMC2323837

[7] Le Gall F , Reusch U , Moldenhauer G , Little M and Kipriyanov SM (2004) Immunosuppressive properties of anti‐CD3 single‐chain Fv and diabody. J Immunol Methods 285, 111–127. 14871540 10.1016/j.jim.2003.11.007

[8] Hudson PJ and Kortt AA (1999) High avidity scFv multimers; diabodies and triabodies. J Immunol Methods 231, 177–189. 10648937 10.1016/s0022-1759(99)00157-x

[9] Holliger P and Hudson PJ (2005) Engineered antibody fragments and the rise of single domains. Nat Biotechnol 23, 1126–1136. 16151406 10.1038/nbt1142

[10] Orita T , Tsunoda H , Yabuta N , Nakano K , Yoshino T , Hirata Y , Ohtomo T , Nezu J , Sakumoto H , Ono K *et al* (2005) A novel therapeutic approach for thrombocytopenia by minibody agonist of the thrombopoietin receptor. Blood 105, 562–566. 15374889 10.1182/blood-2004-04-1482

[11] Kikuchi Y , Uno S , Kinoshita Y , Yoshimura Y , Iida S , Wakahara Y , Tsuchiya M , Yamada‐Okabe H and Fukushima N (2005) Apoptosis inducing bivalent single‐chain antibody fragments against CD47 showed antitumor potency for multiple myeloma. Leuk Res 29, 445–450. 15725479 10.1016/j.leukres.2004.09.005

[12] Fischer‐Colbrie J , Witt A , Heinzl H , Speiser P , Czerwenka K , Sevelda P and Zeillinger R (1997) EGFR and steroid receptors in ovarian carcinoma: comparison with prognostic parameters and outcome of patients. Anticancer Res 17, 613–619. 9066588

[13] Nonomura A , Ohta G , Nakanuma Y , Izumi R , Mizukami Y , Matsubara F , Hayashi M , Watanabe K and Takayanagi N (1988) Simultaneous detection of epidermal growth factor receptor (EGF‐R), epidermal growth factor (EGF) and ras p21 in cholangiocarcinoma by an immunocytochemical method. Liver 8, 157–166. 2839749 10.1111/j.1600-0676.1988.tb00985.x

[14] Salomon DS , Brandt R , Ciardiello F and Normanno N (1995) Epidermal growth factor‐related peptides and their receptors in human malignancies. Crit Rev Oncol Hematol 19, 183–232. 7612182 10.1016/1040-8428(94)00144-i

[15] Reichert JM and Dhimolea E (2012) The future of antibodies as cancer drugs. Drug Discov Today 17, 954–963. 22561895 10.1016/j.drudis.2012.04.006

[16] Keating GM (2010) Panitumumab A review of its use in metastatic colorectal cancer. Drugs 70, 1059–1078. 20481659 10.2165/11205090-000000000-00000

[17] Asano R , Hagiwara Y , Koyama N , Masakari Y , Orimo R , Arai K , Ogata H , Furumoto S , Umetsu M and Kumagai I (2013) Multimerization of anti‐(epidermal growth factor receptor) IgG fragments induces an antitumor effect: the case for humanized 528 scFv multimers. FEBS J 280, 4816–4826. 23890417 10.1111/febs.12451

[18] Asano R , Watanabe Y , Kawaguchi H , Fukazawa H , Nakanishi T , Umetsu M , Hayashi H , Katayose Y , Unno M , Kudo T *et al* (2007) Highly effective recombinant format of a humanized IgG‐like bispecific antibody for cancer immunotherapy with retargeting of lymphocytes to tumor cells. J Biol Chem 282, 27659–27665. 17644522 10.1074/jbc.M704719200

[19] Asano R , Ikoma K , Sone Y , Kawaguchi H , Taki S , Hayashi H , Nakanishi T , Umetsu M , Katayose Y , Unno M *et al* (2010) Highly enhanced cytotoxicity of a dimeric bispecific diabody, the hEx3 tetrabody. J Biol Chem 285, 20844–20849. 20444691 10.1074/jbc.M110.120444 PMC2898345

[20] Asano R , Ikoma K , Shimomura I , Taki S , Nakanishi T , Umetsu M and Kumagai I (2011) Cytotoxic enhancement of a bispecific diabody by format conversion to tandem single‐chain variable fragment (taFv): the case of the hEx3 diabody. J Biol Chem 286, 1812–1818. 21097496 10.1074/jbc.M110.172957 PMC3023476

[21] Nagorsen D and Baeuerle PA (2011) Immunomodulatory therapy of cancer with T cell‐engaging BiTE antibody blinatumomab. Exp Cell Res 317, 1255–1260. 21419116 10.1016/j.yexcr.2011.03.010

[22] Boersma YL and Pluckthun A (2011) DARPins and other repeat protein scaffolds: advances in engineering and applications. Curr Opin Biotechnol 22, 849–857. 21715155 10.1016/j.copbio.2011.06.004

[23] Lofblom J , Frejd FY and Stahl S (2011) Non‐immunoglobulin based protein scaffolds. Curr Opin Biotechnol 22, 843–848. 21726995 10.1016/j.copbio.2011.06.002

[24] Binz HK , Amstutz P , Kohl A , Stumpp MT , Briand C , Forrer P , Grutter MG and Pluckthun A (2004) High‐affinity binders selected from designed ankyrin repeat protein libraries. Nat Biotechnol 22, 575–582. 15097997 10.1038/nbt962

[25] Boersma YL , Chao G , Steiner D , Wittrup KD and Pluckthun A (2011) Bispecific designed ankyrin repeat proteins (DARPins) targeting epidermal growth factor receptor inhibit A431 cell proliferation and receptor recycling. J Biol Chem 286, 41273–41285. 21979953 10.1074/jbc.M111.293266 PMC3308840

[26] Kortt AA , Dolezal O , Power BE and Hudson PJ (2001) Dimeric and trimeric antibodies: high avidity scFvs for cancer targeting. Biomol Eng 18, 95–108. 11566601 10.1016/s1389-0344(01)00090-9

[27] Le Gall F , Kipriyanov SM , Moldenhauer G and Little M (1999) Di‐, tri‐ and tetrameric single chain Fv antibody fragments against human CD19: effect of valency on cell binding. FEBS Lett 453, 164–168. 10403395 10.1016/s0014-5793(99)00713-9

[28] Willuda J , Kubetzko S , Waibel R , Schubiger PA , Zangemeister‐Wittke U and Pluckthun A (2001) Tumor targeting of mono‐, di‐, and tetravalent anti‐p185(HER‐2) miniantibodies multimerized by self‐associating peptides. J Biol Chem 276, 14385–14392. 11278961 10.1074/jbc.M011669200

[29] Arndt KM , Muller KM and Pluckthun A (1998) Factors influencing the dimer to monomer transition of an antibody single‐chain Fv fragment. Biochemistry 37, 12918–12926. 9737871 10.1021/bi9810407

[30] Bayly AM , Kortt AA , Hudson PJ and Power BE (2002) Large‐scale bacterial fermentation and isolation of scFv multimers using a heat‐inducible bacterial expression vector. J Immunol Methods 262, 217–227. 11983235 10.1016/s0022-1759(02)00021-2

[31] Dolezal O , De Gori R , Walter M , Doughty L , Hattarki M , Hudson PJ and Kortt AA (2003) Single‐chain Fv multimers of the anti‐neuraminidase antibody NC10: the residue at position 15 in the V(L) domain of the scFv‐0 (V(L)‐V(H)) molecule is primarily responsible for formation of a tetramer‐trimer equilibrium. Protein Eng 16, 47–56. 12646692 10.1093/proeng/gzg006

[32] Takemura S , Asano R , Tsumoto K , Arai T , Sakurai N , Kodama H , Yoshida H , Katayose Y , Suzuki M , Matsuno S *et al* (2000) Functional Fv fragment of an antibody specific for CD28: Fv‐mediated co‐stimulation of T cells. FEBS Lett 476, 266–271. 10913626 10.1016/s0014-5793(00)01741-5

[33] Fan Z , Lu Y , Wu X and Mendelsohn J (1994) Antibody‐induced epidermal growth factor receptor dimerization mediates inhibition of autocrine proliferation of A431 squamous carcinoma cells. J Biol Chem 269, 27595–27602. 7961676

[34] Yang XD , Jia XC , Corvalan JR , Wang P , Davis CG and Jakobovits A (1999) Eradication of established tumors by a fully human monoclonal antibody to the epidermal growth factor receptor without concomitant chemotherapy. Cancer Res 59, 1236–1243. 10096554

[35] Makabe K , Nakanishi T , Tsumoto K , Tanaka Y , Kondo H , Umetsu M , Sone Y , Asano R and Kumagai I (2008) Thermodynamic consequences of mutations in vernier zone residues of a humanized anti‐human epidermal growth factor receptor murine antibody, 528. J Biol Chem 283, 1156–1166. 17947238 10.1074/jbc.M706190200

